# Paclitaxel Delivery to the Brain for Glioblastoma Treatment

**DOI:** 10.3390/ijms241411722

**Published:** 2023-07-21

**Authors:** Muhammad AbdEl-haq, Awanish Kumar, Fatima-ezzahra Ait Mohand, Nataly Kravchenko-Balasha, Yakir Rottenberg, Abraham J. Domb

**Affiliations:** 1 Institute of Drug Research, School of Pharmacy-Faculty of Medicine, The Hebrew University of Jerusalem, Jerusalem 91120, Israel; 2 The Institute of Biomedical and Oral Research, Faculty of Dental Medicine, The Hebrew University of Jerusalem, Jerusalem 91120, Israelnatalyk@ekmd.huji.ac.il (N.K.-B.); 3 Sharett Institute of Oncology, Hadassah-Hebrew University Medical Center, Jerusalem 91120, Israel; ryakir@hadassah.org.il

**Keywords:** paclitaxel (PTX), poly(lactic-co-glycolic acid) (PLGA), nanoparticles (NPs), brain delivery, intranasal (IN), intravenous (IV), glioblastoma treatment

## Abstract

The development of paclitaxel-loaded polymeric nanoparticles for the treatment of brain tumors was investigated. Poly(lactide-glycolide) (PLGA) nanoparticles containing 10% *w*/*w* paclitaxel with a particle size of 216 nm were administered through intranasal and intravenous routes to male Sprague–Dawley rats at a dose of 5 mg/kg. Both routes of administration showed appreciable accumulation of paclitaxel in brain tissue, liver, and kidney without any sign of toxicity. The anti-proliferative effect of the nanoparticles on glioblastoma tumor cells was comparable to that of free paclitaxel.

## 1. Introduction

The intranasal (IN) delivery route is one of the fastest routes to bypass the blood–brain barrier (BBB) [1]. IN administration is convenient for avoiding the parenteral route and increasing patient compliance. IN absorption enhances drug bioavailability due to its high blood flow, large surface area, and wet environment. The drug is not compromised by gastrointestinal and hepatic pre-systemic metabolism [2]. The presence of a porous endothelial membrane and a highly vascularized epithelium in the nasal cavity provides rapid absorption of the drugs into the systemic circulation [3].

Direct absorption of the drugs into the nasal cavity is possible only with a combination of absorption enhancers. These enhancers, such as phospholipids, salts, and non-ionic surfactants, are materials that can improve mucus solubility and allow the permeation of the materials across the intracellular epithelial barrier [4]. However, direct administration of IN drugs has its own drawbacks. The use of a high amount of absorption enhancers can lead to adverse side effects. Also, processes such as mucociliary clearance and enzymatic degradation increase the local exposure time of the drug and limit the efficiency of drug delivery via IN systems [3]. According to Ugwoke et al. [5], the major toxicological targets for nasally administered drugs include local irritation/tissue damage, epithelial/subepithelial toxicity, and ciliotoxicity.

Nanoscale formulations such as nanoparticles (NPs), nanoemulsions, and nanostructured lipids have been proposed for IN delivery [1] to avoid the local exposure of the drug in the nasal cavity and to increase its bioavailability and stability. We focus, therefore, on the advantages of using biodegradable polymeric NPs (PLGA, polysaccharide, starch, gelatin, etc.) that have been extensively studied because of their unmatched drug encapsulation and release behavior, biocompatible properties, and permeation through the nasal mucosa [6]. It is noted that NPs with sizes ranging from <20 to 500 nm have been reported to absorb in the nasal cavity [7,8]. Brooking et al. [7] examined the effect of particle size (20–1000 nm) on the distribution of polystyrene particles in various organs after nasal administration in a rat model. They found that the particles accumulated in the liver, kidney, spleen, and brain of the animal. Further, based on the comparative observations, Albarki and Donovan [9] indicate that those PLGA NPs with diameters > 100 nm show good uptake into the nasal epithelium and can transfer to the submucosal tissues near the location of significant populations of blood and lymphatic vessels compared to 60 nm NPs. Sonvico et al. [8] point out in their review that a size below 500 nm allows the polymer NPs to squeeze in the non-viscous aqueous pores within the entangled mucin network, further enhancing the interaction with the mucus at a molecular level. Further, Montegiove et al. [10] in their review have provided a list of various polymer NPs with varying sizes that have shown nose-to-brain delivery of therapeutics.

**Figure 1:**
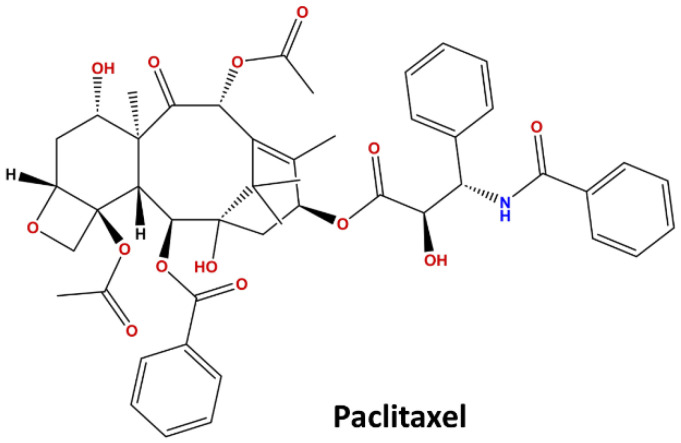
Chemical structure of paclitaxel.

Paclitaxel (PTX) (Figure 1) is widely used as a cytotoxic drug for the treatment of several cancers such as lung, ovarian, and breast cancers, as well as brain tumors. The mechanism of action of PTX interferes with the normal breakdown of microtubules, stopping cell division, which results in cell death [11]. PTX does not dissolve in water or any of the pharmaceutically accepted solvents, hence, it is emulsified using a surfactant and made suitable for the delivery. Taxol is a commercially available formulation containing paclitaxel that is dissolved in a 50:50 *v*/*v* mixture of the surfactant Cremophor EL (polyoxyethylated castor oil) and dehydrated ethanol [12]. Other PTX-based formulations that have been developed or are being tested include albumin-based nanoparticles (e.g., Abraxane), polymeric lipidic nanoparticles (e.g., PICN), polymeric micelles (e.g., Cynviloq, Nanoxel, and Paclical), and liposomes (e.g., Lipusu) [13]. Taxol (generic) and Abraxane (Celgene) are currently two intravenous formulations of PTX that are approved for clinical use in humans; however, both of these formulations lack the self-potential to cross the BBB for use in brain cancer treatment [14]. Fellner et al. [13] and Cruz et al. [15] provide a comprehensive understanding of the studies based on the effects of PTX on glioblastoma (GBM). These studies state that even the soluble form of PTX tested against GBM cannot properly penetrate the BBB. There are very few reports of the IN delivery of PTX in the literature. Cross et al. [16] show that when administered in animals, a very low dose of PTX offers a potential therapeutic option for treating Alzheimer’s disease. Ullah et al. [17] investigated PLGA cancer-targeting arginyl-glycyl-aspartic tripeptide (RGD)-conjugated paclitaxel (PTX)-loaded NPs against GBM using nose-to-brain delivery. RGD-decorated PLGA NPs showed enhanced delivery of PTX to the cancerous area and showed enhanced inhibition of GBM growth [17].

Due to their vascular permeability, polymer NPs can be used for the treatment of glioblastoma (GBM) through the enhanced permeation and retention (EPR) effect [18,19]. As reviewed by Nakamura et al. [20], the EPR effect typically operates in the range of 100–400 nm. However, there is no concrete evidence that describes the relationship between the EPR effect and the size of NPs in humans [21]. The EPR effect can be increased by the local delivery of NPs; however, it comes with a high risk of infection, uncontrolled pH, and osmolarity that if not optimized may lead to brain damage, a significant risk to the patient. Under the given circumstances, the most promising and safest route of administration is the nasal route. Montegiove [10] provided an extensive review listing the promising biocompatible polymer NP carriers for the treatment of glioblastoma (GBM). The authors reviewed and illustrated the drug-loaded polymer NPs that showed promising results in the treatment of GBM; all were in the range of ~160–300 nm. On the other hand, when focusing only on the IN route, the authors showed that polymer NPs with ~40–250 nm size have high accumulation and distribution in the brain [10].

The role of surfactants is also of major concern in the IN delivery of polymer NPs. We shall focus on a new specific kind of non-ionic surfactant, Solutol HS15. Solutol HS15 comprises 70% polyglycol mono- and di-esters of 12-hydroxystearic acid and 30% free polyethylene glycol. It is used as an IN absorption enhancer for drugs [22]. Williams et al. [23] demonstrated the effectiveness of the transmucosal absorption enhancer Solutol HS15 in a nasal spray formulation using a preclinical pharmacokinetic model for the drug PTH 1–34. Similarly, Zhang et al. [24] also confirmed that Solutol HS15 can promote intranasal absorption of the drug nalmefene hydrochloride (NMF). Moran et al. [25,26] developed a possible method of encapsulating water-soluble drugs in polymeric NPs that are most suitable for IN delivery. This study is focused on the pharmacokinetics and biodistribution of PTX-loaded PLGA NPs, administered to rats by IN and IV delivery.

## 2. Results and Discussion

### 2.1. In Vitro Release

A solvent evaporation method was used for the preparation of 10% PTX-loaded PLGA NPs at 90% yield. Table 1 contains the details of the characterization of the NPs. The PTX-loaded NPs were also checked for stability when stored at −20 °C. No crystallization of the PTX was seen in the NPs upon cold storage.

**Figure 2:**
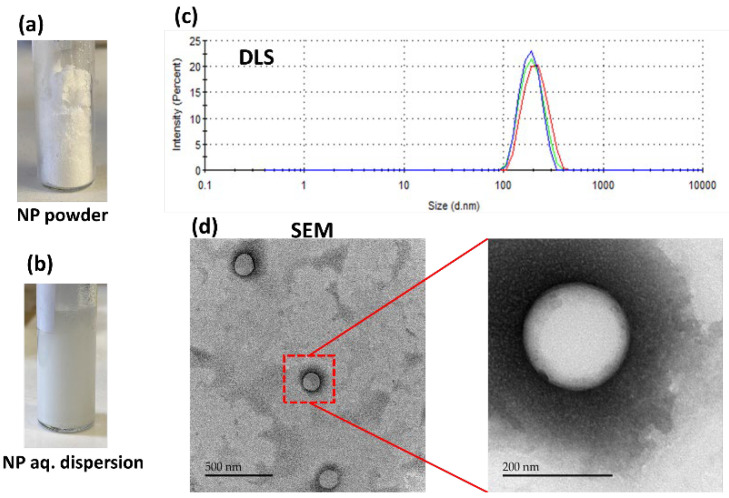
PTX-loaded PLGA NPs: (**a**) NP powder; (**b**) aqueous dispersion of NPs; (**c**) DLS analysis of aqueous dispersion of NPs. The samples were tested in triplicate indicated as three colors; (**d**) TEM scanning of the NPs.

**Figure 3:**
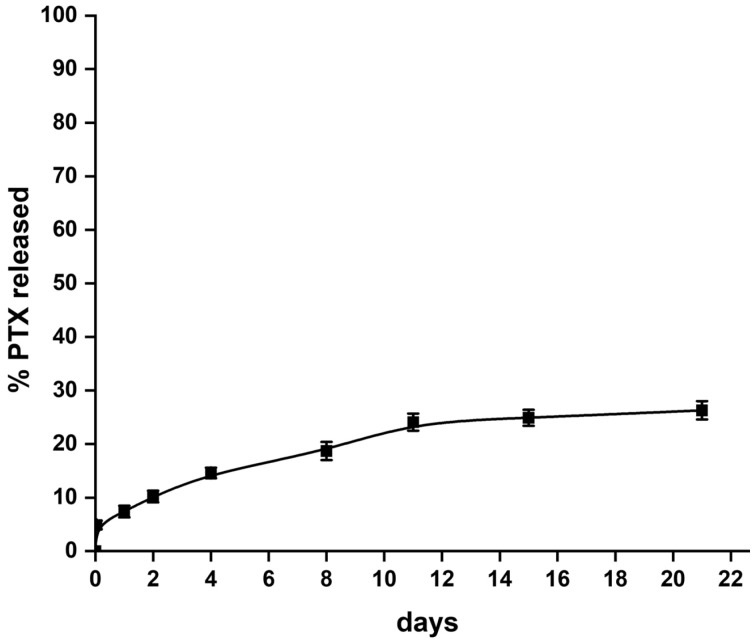
Release of PTX from the PLGA NPs in 1% Tween 80 at 37 °C.

As shown in Figure 2, PTX-loaded NPs were obtained as a dry powder that easily disperses in DDW. Particles of 216 ± 0.8 nm with a polydispersity index (PDI) of 0.194 ± 0.02 were obtained, indicating uniform polydispersity [27]. The release of the PTX from the NPs was monitored in PBS containing 1% *w*/*v* of Tween 80 (Figure 3). Tween 80 was used to ensure sink conditions due to PTX’s limited solubility in water, less than 0.1 μg/mL [28]. A 30% cumulative release of PTX in 20 days was observed, which is similar to previous reports [29,30].

### 2.2. Pharmacokinetic Analysis

The plasma analysis of the PTX after intranasal (IN) and intravenous (IV) administration of the PLGA NPs was determined. The selection of the PTX dose of 5 mg/Kg was based on the study by Shord and Camp [31]. No side effects or abnormal behavior was observed in the animals after both IN and IV delivery of the PTX-loaded NPs.

**Figure 4:**
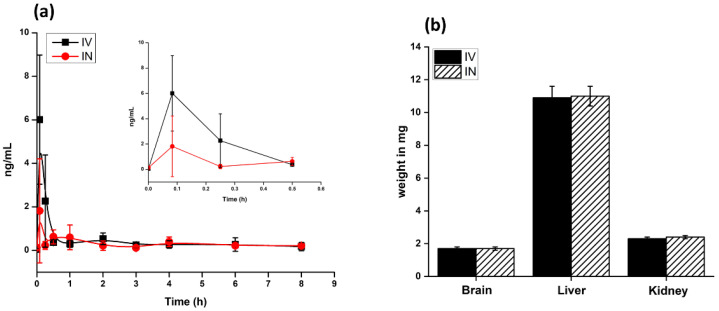
(**a**) PTX quantification in blood plasma after IN and IV administration of the PLGA NPs. Inset provided is the PTX concentration observed within 30 min of administration. (**b**) Weights of the various organs after the IN and IV administration of the PLGA NPs.

The average weights of the rats were not changed after the administration of the PTX-loaded PLGA NPs. The average weights of the animals throughout the study were 282 ± 6 and 278 ± 9 g for IV and IN administration, respectively. No side effects in the animal behavior were observed at any time point in the study. The organs were collected at the end of the experiment and examined visually for any sign of toxicity or irregular tissue appearance. No changes in the visual appearance or weight of the organs from the animals receiving IN and IV administration were seen, compared to the untreated control group. As shown in Figure 4, the average weights of the brain, liver, and kidney for the animals treated with IV NPs were 1.7 ± 0.1 g, 10.9 ± 0.7 g, and 2.3 ± 0.1 g, respectively. Similarly, the average weights of the brain, liver, and kidney for the animals that received IN NPs were 1.7 ± 0.1 g, 11.0 ± 0.6 g, and 2.4 ± 0.1 g, respectively. The average weight of the animals, as well as their organs, for the control group will presumably be similar.

The plasma PTX concentration profiles following IN and IV administration are shown in Figure 4, and data are provided in Table 2. PTX was rapidly absorbed into the systemic circulation following IN and IV administration of the NPs with a T_max_ of 5 min. The AUC_0–24_ plasma values after 5 min of IN and IV administration of PTX were 2.6 and 3.9 ng·h/mL, respectively.

The plasma concentration of the PTX is seen within the 5 min of IN administration of the NPs, which indicates that most NPs avoid mucociliary clearance. According to previous reports [32,33], mucociliary clearance is the primary physiological mechanism that significantly limits nose-to-brain transport. The authors found no significant differences in mucociliary clearance of the type of particle under investigation. It is proposed that when the particles that lack significant diffusivity are trapped in the mucus, they clear at a velocity equal to the clearance velocity of the mucus, independent of particle properties [33]. A similar mechanism can be suggested in our case. Upon administration, most of the NPs are trapped in the IN mucosa, and only <1% of the drug reaches the brain [34]. Another factor is the procedure of intranasal administration in rats. In general, IN delivery of an aqueous dispersion of NPs involves using a pipette tip to form a small droplet in the nostril of the animal. Since a very low volume of an NP dispersion is used for nasal administration, it is unlikely that all the NPs will be distributed in the nasal cavity for the required concentration of the drug to be delivered to the brain [35,36,37].

Based on pharmacokinetic analysis and comparison with the IV administration of the PTX-loaded PLGA NPs, a significant amount of PTX can be seen in the brain. This indicates that whatever amount of the NPs administered intranasally reaches the trigeminal nerves present in the respiratory and olfactory regions is delivered directly to the brain. In addition, those NPs that enter the systemic circulation due to mucociliary clearance can pass through the airway or esophagus and are directly delivered to the brain [38]. On the other hand, the intravenously administered NPs also show an accumulation of PTX in the brain tissues. This indicates the possible penetration of the developed NPs through the BBB, while PTX does not cross the BBB [13,39,40]. The penetration of PLGA NPs into the brain after intravenous delivery has already been reported [41,42,43,44]. In these studies, PLGA NPs were used to deliver camptothecin [42], doxorubicin [43], and curcumin [44] to the brain.

**Figure 5:**
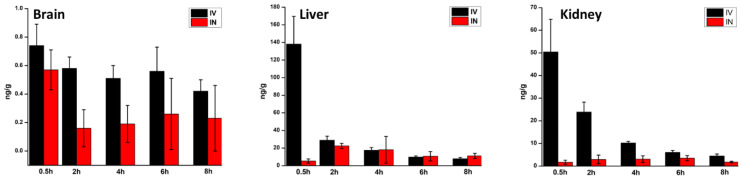
PTX distribution in organs after IN and IV delivery. The dose of the PTX in both IN and IV was 5 mg/kg.

As shown in Figure 5, at the 0.5 h time point after IV administration, highly perfused systemic tissues such as the liver and kidney showed relatively high concentrations of PTX. At this time point (0.5 h), the PTX accumulation in the tissues compared to the IN administration of the NPs tended to be high for the same dose of PTX. The maximum PTX concentration in the liver and kidney after IN administration of the NPs is achieved at 2 h and 6 h, respectively. As presented in Table 2, the AUC_0–24_ values of PTX in the liver and kidney after IN administration are 113.5 and 22.0 ng·h/mL, respectively. These facts suggest that the mucociliary clearance of the PTX-loaded PLGA NPs is slower, and the NPs are slowly transferred to the systemic circulation from the nasal cavity after IN administration. On the other hand, AUC_0–24_ of PTX in the liver and kidney after IV administration is 274.9 and 141.3 ng·h/mL, respectively. The IV administration indicates more PTX accumulation in the liver and kidneys of the rats, because of the systemic circulation of the NPs in the body that is distributed to the liver and kidney, where it will be metabolized and eliminated. The terminal half-life of PTX was calculated at about 2 h in the liver and kidney following the IV route. The PTX half-life in the rest of the tissues was not accepted as reliable. The tissue penetration of PTX in the NP formulations was measured by calculating the ratio of PTX concentrations in tissues versus plasma at T_max_. The ratio of the distribution of PTX in the various tissues compared to plasma was in the ranges of 0.07 to 7.4 (C_max_, IV), 0.3 to 12.4 (C_max_, IN), and 1.1 to 70.5 (AUC_0–24_, IV), 0.8 to 43.6 (AUC_0–24_, IN), as seen in Table 2.

### 2.3. Viability Study

**Figure 6:**
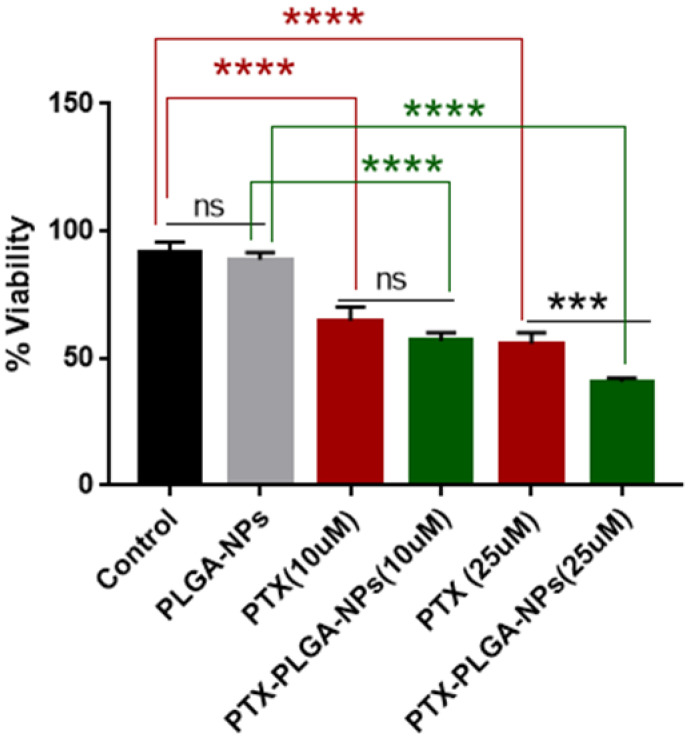
PTX-loaded nanoparticles are more effective than PTX alone in reducing the viability of GBM cells. Cell viability was determined by performing an MTT assay on U87MG cells 48 h after treatment with the indicated PTX concentrations. Statistical analyses of mean ± SD (standard deviation) were performed using Prism GraphPad 9.5.1.733. *** *p* < 0.0005, **** *p* < 0.0001, ns: not significant.

The MTT cell proliferation assay was performed using a U87MG cell line harboring the amplification of the epidermal growth factor receptor (U87EGFRwt) to evaluate the anti-proliferative effect of PTX-loaded PLGA NPs on GBM cells. The cells were exposed to blank PLGA NPs (360 mg/mL) and PTX or PTX-loaded PLGA NPs at two different concentrations for 48 h (10 μM or 25 μM). Cell viability after 48 h was comparable between the control and PLGA-NP-treated cells, and when cells were treated with 10 µM of free PTX or PTX-loaded PLGA NPs. However, at 25 µM, PTX-loaded PLGA NPs significantly outperformed PTX alone in terms of anti-proliferative activity against GBM cells (Figure 6). This suggests that PLGA-NPs provide sustained release of encapsulated PTX, potentially improving cellular uptake.

The ability of NPs to penetrate the BBB depends not only on the NPs’ size but also on the charge and surface properties of the particles [45,46,47]. Li and Sabilov [48] noted that the uptake of the polymer NPs into the brain can be enhanced when the surface of the NPs is modified using suitable surfactants. Interestingly, Nowak et al. [49] observed a nonmonotonic dependence on polystyrene particle size, where 200 nm particles exhibited higher BBB transport compared to 100 and 500 nm spheres. Kulkarni and Feng [50] also showed that the TPGS-coated polystyrene NPs of 200 nm size have the potential to deliver the drug across the BBB. Thus, the availability of our developed PTX-loaded PLGA NPs in the brain tissue can be attributed to the significant contribution of Solutol HS15.

Overall, the PTX-loaded PLGA NPs did show significant PTX levels in the brain, liver, and kidney after IV and IN administration. The ability of lactide-containing polymers such as polylactide (PLA) NPs to cross the BBB was proven by Kubek et al. [51]. Using the kindling model of temporal lobe epilepsy and fluorescence imaging techniques, the authors showed that the drug-loaded polymer NPs can effectively cross the BBB and suppress seizures and perhaps epileptogenesis [51]. Significantly, the developed PTX-loaded PLGA NPs can pass the BBB when administered intravenously. In the long run of developing a PLGA-based NP system, suitable for both IN and IV administration, the developed PTX-loaded PLGA NPs using Solutol HS15 as a surfactant have shown their potential application. The combined effect of IV and IN administration of our developed PLGA-based NPs can be advantageous for controlling and treating brain tumors in the future.

## 3. Materials and Methods

### 3.1. Materials

Poly(lactide-co-glycolide) (PDLG 5010) (50:50) M_w_ 14 kDa was purchased from Corbion Purac, Amsterdam, The Netherlands. Paclitaxel (PTX) was obtained from Bioxel Pharma Inc., Quebec, QC G1V 0B6, Canada. Solutol HS15 was kindly gifted by BASF. Docetaxel was purchased from Sicor de México, Toluca, México. Acetonitrile (HPLC grade) and ethanol (HPLC grade) were obtained from BioLab, HaYetsira, Jerusalem, Israel. Pluronic F127 and Tween 80 were purchased from Sigma Aldrich, St. Louis, MO, USA.

### 3.2. Method

#### 3.2.1. Nanoparticle (NP) Preparation

The NPs were prepared using the antisolvent method by dissolving 50 mg of PTX and 500 mg of PLGA into 10 mL of acetonitrile. The organic solution was added dropwise into 50 mL 0.2% *w*/*v* of Solutol HS15 aqueous solution and stirred at 1000 rpm for 1 h. Thereafter, acetonitrile was evaporated using a rotavapor. The NPs were then collected by centrifuging the aqueous dispersion at 7000 rpm and 4 °C for 50 min. The supernatant was removed carefully, and the NPs were washed again with 15 mL of double-distilled deionized water (DDW) and collected again by centrifuging at 7000 rpm and at 4 °C for 50 min to remove excess surfactant. It should be noted that centrifuging the dispersion at 7000 rpm and at 4 °C for 50 min helps to obtain a redispersible NP pellet. Further, the obtained NP pellet was redispersed in 2 mL of 1% *w*/*v* Pluronic F127 aqueous solution and lyophilized. The NPs were stored at −20 °C in air-tight glass vials until use.

#### 3.2.2. PTX Quantification

- High-performance liquid chromatography (HPLC)

PTX 1 mg/mL concentration was prepared in ethanol to obtain the standard curve. Then, 1 mL of the methanol solution was transferred into 19 mL of 1% *w*/*v* Tween 80 solution in phosphate buffer solution (PBS, pH 7.2 ± 0.2) to prepare the stock solution of 50 µg/mL. Using the stock solution of 50 µg/mL, 1–50 µg/mL concentrations of PTX in PBS with 1% *w*/*v* Tween 80 were prepared. Standards solutions were filtered using a 0.45 µm filter and injected into the HPLC.

Analysis was performed on a LiChrospher 100 RP-18 (5 µm) column packed in a LiChoCart 250-4 HPLC cartridge (Merck KGaA, Darmstadt, Germany). The chromatographic system used was a Merck Hitachi Lachrom HPLC system equipped with a UV detector (Model Lachrom L7400). The mobile phase consisted of acetonitrile:DDW (60:40), used in isocratic mode. The sample injection volume was 20 µL. The analysis was carried out at 25 °C at a flow rate of 1.0 mL/min. The effluent was monitored on a UV detector attached to the HPLC system at a wavelength of 229 nm. PTX showed a retention time of 5.4 ± 0.2 min under these conditions. Calibration plots were prepared in a concentration range of 1–50 μg/mL.

b. Liquid chromatography–mass spectrometry (LC-MS)

MS/MS analyses were conducted on a Sciex (Framingham, MA, USA) QTRAP 6500+ mass spectrometer. Air was produced (SF 4 FF compressor, Atlas Copco, Antwerpen, Belgium) and purified using an Infinity 1031 nitrogen generator (Peak Scientific, Inchinnan, Scotland). Purified air was used as source and exhaust gases, and purified nitrogen was used as curtain and collision gases. A receiver was placed between the compressor and the nitrogen generator for a large and stable supply of air. Chromatography was performed under reverse-phase conditions using a Shimadzu (Kyoto, Japan) UHPLC system, consisting of a Shimadzu CBM-20A communication bus module, Nexera X2 LC-30AD pump, a Shimadzu DGU-20A5R degasser, a Shimadzu SIL-30AC autosampler, and a Shimadzu CTO-20A column oven. Liquid chromatographic separation was obtained using 5 μL injections of samples onto a Kinetex 2.6 μm C18 (100 × 2.1 mm) column from Phenomenex (Torrance, CA, USA). The autosampler was set at 15 °C, and the column was maintained at 40 °C during the entire analysis. Data acquisition was performed on a Dell Optiplex XE2 computer using Analyst 1.7.1, and data were analyzed using Sciex OS Software version 3.2. Gradient elution mobile phases consisted of 0.1% formic acid in water (phase A) and 0.1% formic acid in methanol (phase B). Gradient elution (350 μL/min) was held at 2% B for the first 1 min, followed by a linear increase towards 40% B in 2 min and held at 40% B for 1 min, then followed by a linear increase towards 85% B in 5 min and held at 85% B for 2.5 min. Paclitaxel was detected in positive ion mode using electron spray ionization (ESI) and the multiple reaction monitoring (MRM) mode of acquisition, using docetaxel as an internal standard (IS). The IonDriveTM Turbo V source temperature was set at 650 °C with the ion spray voltage at 5500 V. The curtain gas was set at 20.0 psi. The nebulizer gas (Gas 1) was set to 30 psi, the turbo heater gas (Gas 2) was set to 20 psi, and the dwell time was 30 ms. Collision energy (CE), declustering potential (DP), and collision cell exit potential (CXP) for the monitored transitions are given in Table 3.

c. Determination of drug loading and entrapment efficiency

The amount of free PTX was determined by adding 10 mg of the dry NP powder into a glass vial with 1 mL ethanol and shaking the vial for 10 min. The sample was centrifuged at 13,500 rpm for 20 min, and the clear supernatant was injected into the HPLC. The total drug loading was determined by adding 10 mg of the NP powder to 1 mL ACN in a 2 mL glass vial. The vial was placed on a vortex to fully dissolve the particles. Then, 0.5 mL ethanol was added to precipitate the polymer, and the solution was centrifuged at 13,500 for 10 min. A clear supernatant was obtained, which was filtered with a 0.22 µm PTFE filter and examined for the total drug obtained using the developed HPLC method. True drug loading in the NPs was determined by subtracting the free drug obtained by washing the NPs in ethanol from the total drug obtained using acetonitrile.

d. Particle size and surface charge

Dynamic light scattering (DLS) was performed to determine the size of the PTX-loaded PLGA NPs using a Zetasizer (Nano ZS, Malvern Instruments, Malvern WR14 1XZ, UK) equipped with inbuilt software. Nearly 1 mL of the NP dispersion in DDW was placed into a glass cuvette (12 mm glass cell with square aperture, cell type: PCS1115). NPs were appropriately diluted with the applicable amount of DDW. The measurement angle was selected at 173° backscatter (NIBS) in the instrument’s automatic selection mode. The z-average of the NPs was calculated using the auto-correlation function of the intensity of light scattered from the particles, assumed to be in the spherical form by the instrument software. All measurements were conducted in triplicate at 25 °C for the NPs.

e. Particle morphology

Transmission electron microscopy (TEM) analysis was performed using a JEOL JEM-1400Plus by applying ~10 μL of samples resuspended in DDW to a 200- or 400-mesh copper grid covered by carbon-stabilized Formvar film (SPI, West Chester, PA, USA). The samples were dried overnight before scans were performed. The NPs were sequentially stained with negative stain NanoVan (Methylamine Vanadate) (Nanoprobes, Inc., Yaphank, NY, USA), and the images were analyzed using inbuilt software (SoftImaging System GmbH, Münstar, Germany).

f. In vitro drug release

The in vitro release study was conducted by transferring 10 mg of the nanoparticles into an Eppendorf vial with 2 mL of PBS pH 7.2 containing 1% *w*/*v* Tween 80 at 37 °C under constant shaking at 75 rpm. The release medium was replaced periodically with a fresh buffer after centrifuging at 13,000 rpm for 15 min, and PTX content in the solution was determined using the HPLC method.

### 3.3. Cell Culture and Treatment

U87EGFRwt cell lines were created by modifying the U87 human glioblastoma cell line to express wtEGFR (U87EGFRwt). This was discovered to be consistent with the elevated EGFR levels commonly found in GBM tumors prepared from patient material [52]. The cell lines were authenticatssed by the Genomic Center of the Technion Institute (Haifa, Israel) and checked on a routine basis for the absence of mycoplasma. Cell lines were routinely maintained as described [53].

Glioblastoma cells were seeded in 96-well plates at a density of 3 × 10^4^ cells/well and then treated for 48 h with either free PTX (PTX, dissolved in DMSO) or equivalent PTX-loaded PLGA-NPs at two different concentrations (10 μM or 25 μM). At all concentrations tested, PTX was completely dissolved in cell culture media.

**Survival assay**: In a 96-well plate, cells were seeded and treated as directed for 48 h. The MTT assay kit (#ab211091, Abcam, Cambridge, UK) was used to assess cell viability. Each well received equal volumes of MTT solution and culture media, and it was incubated at 37 °C for 3 h. After adding MTT solvent to each well, the plate was covered with aluminum foil and placed on an orbital shaker for 15 min. Within 1 h, the absorbance at 590 nm was measured.

### 3.4. Animal Study

All animal studies were performed in the standard facilities of Pharmaseed. Pharmaseed is Israel’s largest GLP-certified preclinical and early clinical CRO, specializing in translational and regenerative studies (www.pharmaseedltd.com; accessed on 17 July 2023). Eight-week-old male Sprague–Dawley (SD) rats weighing 278.3 ± 9.2 g were used in this study.

Blood samples were collected from male Sprague–Dawley (SD) rats to determine plasma concentrations of PTX. Thirty SD rats per route of administration were used for this study for the test item. The procedures included one (1) test item (Taxol) at one (1) dose level (5 mg/kg/day) in two (2) formulations for intravenous (IV) and intranasal (IN) administrations.

Test items were administrated to each animal once. Plasma sampling times were pre-dose and 5, 15, and 30 min and 1, 2, 3, 4, 6, and 8 h after test item dosing. Tissue sampling times were 30 min and 2, 4, 6, and 8 h after test item dosing.

#### 3.4.1. Pharmacokinetic (PK) Study

The pharmacokinetic (PK) profile and distribution of paclitaxel nanoparticles (NPs) were evaluated following single intranasal (IN) or intravenous (IV) administration in 8-week-old male SD rats. The rats were randomly assigned to either the IV administration group (n = 15) or the IN group (n = 15). The IV group was administrated with 5 mg/kg with a total volume of 1 mL/kg. The IN administration was at the same dose of 5 mg/kg with a total volume of 0.2 mL/kg. Following administration, blood samples were collected at predetermined timepoints (0, 5, 15, 30, 60, 120, 180, 240, 360, and 480 min); after the last bleeding for each subgroup, the animals received an SC injection of buprenorphine 0.1 mg/kg on a heating pad and were anesthetized with a ketamine/xylazine mixture, and perfusion was performed to sacrifice the animals. The brain, liver, and kidney tissues were collected, weighed, and stored at −80 °C until further analysis.

#### 3.4.2. LC-MS Calibration Curve for PTX in Blood Plasma

The calibration curve in blood plasma was prepared by adding the appropriate amount of PTX and DTX to 500 µL of plasma. The sample was placed on a vortex for 20 min, and then the appropriate amount of ACN was added to achieve the desired PTX concentration in a total volume of 2 mL. The samples were centrifuged at 13,500 rpm for 15 min at 4 °C, and the supernatant was transferred into a new vial for LCMS. The PTX concentrations were prepared in the range of 0–250 ng/mL. The DTX concentration in all the samples was 250 ng/mL.

#### 3.4.3. PTX Bioanalysis

- **Blood Analysis**: A plasma aliquot (100 µL) was spiked with 10 µL of IS solution (DTX—250 ng/mL) and mixed with 290 µL of ACN. The final samples were centrifuged for 15 at 13,500 rpm and 4 °C to precipitate the plasma proteins. The clear supernatant was transferred into appropriate LCMS vials after filtration through 0.22 µm PTFE filters. Specimens of the brain, liver, and kidney were dissected free of surrounding tissue, blotted free of fluid, and weighed. The tissue samples were then homogenized in 2 mL of acetonitrile, using a homogenizer. The homogenate was centrifuged at 13,500 and 4 °C for 15 min, and 390 µL of the supernatant was spiked with 10 µL of IS solution (DTX—250 ng/mL) and transferred into new vials after filtration through a 0.22 µm PTFE filter. The extraction was then examined using the developed LCMS method. The stated procedure for obtaining the PTX from the blood samples and tissues was confirmed by extracting the drug from the NPs. The NPs were dissolved, and the PTX concentration was tested. One hundred percent of the PTX was recovered from the NPs, which supports the developed extraction procedure.
- **Pharmacokinetic (PK) analysis**: Pharmacokinetic (PK) parameters were calculated using the computer program PK Solutions 2.0 (Summit Research Services, Portland, OR, USA). All plasma and tissue concentrations supplied as part of this study were reported to two decimal places. Pharmacokinetic parameters were reported as follows: C_max_, AUC_0–t_, and AUC_inf_ with no decimal places; λ_Z_ to 3 decimal places; t_1/2_ to 1 decimal place. Standard deviations were reported to the same precision as the corresponding mean value. Unless stated otherwise, all summary statistics (e.g., mean, SD) presented in this report are based on the rounded numbers shown in the tables. If BLQ values represented 50% or more of the total number of observations at any time point, means were not calculated; if the mean value was BLQ, then it was not presented in the report. To facilitate a logarithmic transformation of the data, values recorded as BLQ were depicted as half of BLQ (0.01). Values that were above ULOQ (>250 ng/mL) were entered as 250 ng/mL in the calculation of the mean.

Maximum observed plasma concentrations (C_max_) and their times of occurrence (T_max_) were the observed values. Areas under the plasma concentration–time curves up to the last quantifiable concentration (AUC_0–t_) were calculated using the linear trapezoidal rule. In the calculation of AUC_0–t_ values, it was assumed that the pre-dose (0 h) plasma concentrations were zero. AUC_inf_ was calculated using by combining AUC_0–t_ with an extrapolated value (Cn/λ_Z_). Data permitting, the terminal elimination rate constant (λ_Z_) was estimated by fitting a linear regression of log concentration against time, and t_1/2_ was calculated as ln2/λ_Z_. For the estimate of λ_Z_ to be accepted as reliable, the following criteria were imposed:

1. The terminal data points were apparently randomly distributed about a single straight line (on visual inspection).
2. A minimum of 3 data points were available for the regression.
3. The regression coefficient was ≥0.85.
4. The interval including the data points chosen for the regression was at least 2-fold greater than the half-life itself.

## 4. Conclusions

Paclitaxel-loaded PLGA nanoparticles delivered intranasally or intravenously were detected in the rat brain as well as in the liver and kidney. No local or systemic toxicity or change in animal behavior was noted. This is attributed to the complete encapsulation and controlled release of the paclitaxel from the PLGA NPs in an aqueous medium. The results of this study indicate brain delivery of the developed NPs as noted by Li and Kataoka [19]. The simultaneous IN and IV co-administration of the developed NPs can be advantageous in brain tumor treatment, which needs further studies.

## Figures and Tables

**Table 1:** Physical characterization of the PLGA NPs.

PTX-Loaded PLGA NPs	Characterization
Drug loading	9–10% *w*/*w*
Loading efficiency	~98%
Particle size (DLS)	216 ± 0.8 nm
PDI (DLS)	0.194 ± 0.02
Particle size (TEM)	191.9 ± 10.7 nm
Morphology	Spherical shape
Surface charge	−16 ± 2 mV

**Table 2:** Pharmacokinetic parameters of PTX following IN and IV administration to male SD rats.

	T_max_ (h)	C_max_ (ng/mL)	AUC_0–24_ (ng·h/mL)	AUC_inf_ (ng·h/mL)	t_1/2_ (h)	λ_Z_ (1/h)
**Taxol (dose 5 mg/kg)—IV**						
n	3	3	3	3	3	3
Plasma	0.083	10.13	3.9	NA	NA	NA
Brain	0.5	0.74	4.5	NA	NA	NA
Liver	0.5	74.5	274.9	297.4	2.0	0.351
Kidney	0.5	50.5	141.3	155.2	2.1	0.324
**Taxol (dose 5 mg/kg)—IN**						
n	3	3	3	3	3	3
Plasma	0.083	1.82	2.6	NA	NA	NA
Brain	0.5	0.57	2.0	NA	NA	NA
Liver	2.0	22.5	113.5	NA	NA	NA
Kidney	6.0	3.53	22.0	NA	NA	NA

NA—λ_Z_ estimate was not accepted as reliable (see imposed criteria).

**Table 3:** Optimized multiple reaction monitoring (MRM) transitions.

Compound	Molecular Ion [M + Na]^+^ (*m*/*z*)	Fragment (*m*/*z*)	DP (Volts)	CE (Volts)	CXP (Volts)	Retention Time (min)
**Paclitaxel**	876	308 (quantifier)	100	40	20	11.1
		591 (qualifier)	100	35	10	
**Docetaxel**	830	549 (quantifier)	1	35	16	10.9
		304 (qualifier)	1	31	26	

## Data Availability

The datasets used and/or analyzed during the current study are available from the corresponding author upon reasonable request.
